# Intracellular Habitation of *Staphylococcus aureus*: Molecular Mechanisms and Prospects for Antimicrobial Therapy

**DOI:** 10.3390/biomedicines10081804

**Published:** 2022-07-27

**Authors:** Josefien W. Hommes, Bas G. J. Surewaard

**Affiliations:** Department of Microbiology, Immunology and Infectious Diseases, Snyder Institute for Chronic Diseases, Cumming School of Medicine, University of Calgary, Calgary, AB T2N 4N1, Canada; josefien.hommes@ucalgary.ca

**Keywords:** *Staphylococcus aureus*, MRSA, intracellular infection, antibiotic resistance, antimicrobial therapy

## Abstract

Methicillin-resistant *Staphylococcus aureus* (MRSA) infections pose a global health threat, especially with the continuous development of antibiotic resistance. As an opportunistic pathogen, MRSA infections have a high mortality rate worldwide. Although classically described as an extracellular pathogen, many studies have shown over the past decades that MRSA also has an intracellular aspect to its infectious cycle, which has been observed in vitro in both non-professional as well as professional phagocytes. In vivo, MRSA has been shown to establish an intracellular niche in liver Kupffer cells upon bloodstream infection. The staphylococci have evolved various evasion strategies to survive the antimicrobial environment of phagolysosomes and use these compartments to hide from immune cells and antibiotics. Ultimately, the host cells get overwhelmed by replicating bacteria, leading to cell lysis and bacterial dissemination. In this review, we describe the different intracellular aspects of MRSA infection and briefly mention *S. aureus* evasion strategies. We discuss how this intracellular niche of bacteria may assist in antibiotic tolerance development, and lastly, we describe various new antibacterial strategies that target the intracellular bacterial niche.

## 1. Introduction

As the COVID-19 pandemic continues, it represents the leading cause of morbidity and mortality worldwide, causing over 6 million deaths to date. In addition to the human toll, this pandemic represents the single largest burden on healthcare and the world economy. In the lee of this global pandemic, bacterial infections continue in the shadows. Bacterial antimicrobial resistance (AMR) occurs when changes in microbes cause the antimicrobials used to treat infections to become ineffective, and this has emerged as one of the leading public health threats of the 21st century. A recent systematic review showed an estimated 4.95 million annual infections associated with bacterial AMR in 2019, which caused 1.27 million deaths [1]. By far the most dangerous drug-resistant pathogen in 2019 was methicillin-resistant *Staphylococcus aureus* (MRSA) causing over 100,000 deaths alone. This is almost double compared to mortality caused by other ESKAPE pathogens, such as *Klebsiella pneumoniae*, *Streptococcus pneumoniae*, *Acinetobacter baumannii*, and *Pseudomonas aeruginosa* [1]. What sets MRSA apart is its tremendous reservoir of virulence factors and immune evasion molecules that in combination with AMR make it a particularly deadly pathogen.

*Staphylococcus aureus* (*S. aureus*) is part of the normal skin and nasal microbiota, with approximately 30% of the healthy adult population colonized mainly in the nasopharyngeal cavity. While colonization is usually asymptomatic, a symptomatic infection can occur if there is a breach in the mucosal barrier or skin. The severity of symptomatic infections ranges from superficial skin and soft tissue infections [2], to devastating complications, such as necrotizing pneumonia, endocarditis, toxic shock syndrome, and sepsis [3,4]. In the pre-antibiotic era, *S. aureus* bacteremia mortality rates were astonishingly high, ranging between 75% and 83% [5]. Even though antibiotics have reduced this number significantly, *S. aureus* bloodstream infections still account for over 19,000 deaths annually in the United States [6,7]. With every new antibiotic that is developed, *S. aureus* resistance has been quickly observed [8]. MRSA strains that are resistant to all penicillin-like β-lactam antibiotics pose a particularly serious threat to the community [9]. Two types of MRSA exist: hospital-acquired (HA-)MRSA and community-acquired (CA-)MRSA [10]. CA-MRSA strains are typically regarded as more virulent and can cause infections in otherwise healthy individuals. This notion is further supported by experimental animal studies, whereas HA-MRSA strains are less virulent than CA-MRSA and cause fewer disseminating diseases. Although in animal models, mice are typically not treated with antibiotics, which is unlike a hospital setting [11]. 

The standard treatment for systemic MRSA infections in most countries is 2–6 weeks of intravenous vancomycin administration, which is one of the last remaining treatment options. Vancomycin-resistant *S. aureus* (VRSA) strains have been reported; however, these infections are still unlikely to occur [12]. Hence, most MRSA infections remain susceptible to vancomycin. Nonetheless, relapse of MRSA infections is quite common, even after prolonged antibiotic treatment of patients [13]. These data suggest the existence of a staphylococcal reservoir that is tolerant to vancomycin treatment. This phenomenon was also observed in experimental mouse studies in which infections with vancomycin-susceptible MRSA strains could still cause disease even in the presence of vancomycin [14]. State-of-the-art in vivo imaging revealed that this may be due to *S. aureus* adapting to intracellular habitation in macrophages, where bacteria are sheltered from the harmful effects of antibiotics. Upon termination of the vancomycin treatment, the escaped bacteria could survive in the extracellular microenvironment and cause dissemination to other organs and sepsis [15].

Most microbiology and infectious disease textbooks state that *S. aureus* is solely an extracellular pathogen, however, over the last decades, there has been a significant body of evidence showing that *S. aureus* can have a pronounced intracellular component in its pathogenic lifecycle [16]. During infection, *S. aureus* actively employs several invasion strategies to hide inside non-immune cells and expresses an abundance of virulence factors that combat antimicrobial effector functions of phagocytes [17]. Despite *S. aureus*’ extensive immune evasion repertoire, which includes many molecules to evade uptake by phagocytes, the bacteria are inevitably phagocytosed by professional phagocytes such as macrophages or neutrophils [18]. Within these professional phagocytes, *S. aureus* ends up in phagolysosomes, which are bactericidal compartments with the main purpose of eradicating bacteria. However, it has become clear that *S. aureus* can withstand the killing mechanisms of professional phagocytes and survive intracellularly [15,19,20,21,22]. Evidence suggests that the intracellular survival of *S. aureus* plays a critical role in the antibiotic tolerance of these bacteria. In this review, we will discuss the virulence mechanisms that mediate the intracellular survival of *S. aureus*, together with the current development of antibiotic therapies that specifically target the intracellular *S. aureus* reservoir.

## 2. The Infectious Cycle of *S. aureus* in Non-Professional Phagocytes

**Figure 1:**
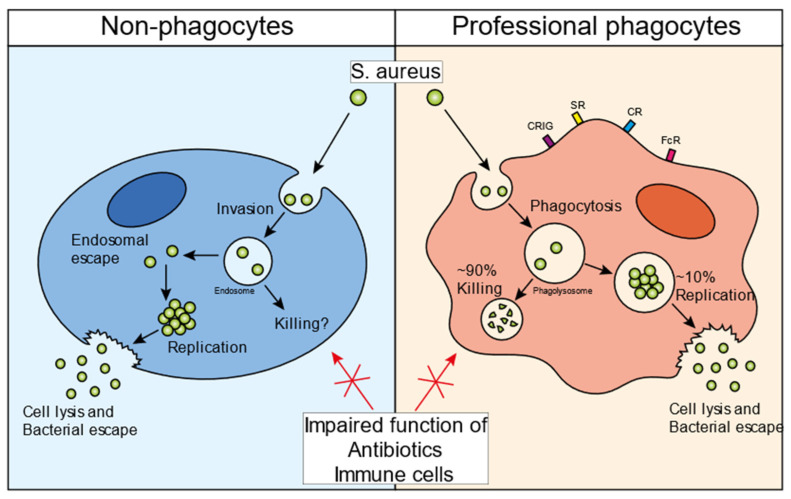
*S. aureus* infectious cycle. Schematic overview of the staphylococcal infectious life cycle in non-professional phagocytes (**left panel**) and professional phagocytes (**right panel**). *S. aureus* invades non-professional phagocytes and ends up inside the endosome. Various bacterial factor regulators, such as Agr and SigmaB, are important for the endosomal escape of the bacteria into the cytosol. Once inside the cytosol, bacteria can persist inside the host cells, maintaining an intracellular bacterial reservoir. Additionally, bacteria can start replicating, eventually lysing the host cell, and escaping into the extracellular environment. Professional phagocytes recognize bacteria with different receptors and take up bacteria by means of phagocytosis (**right panel**). Once inside the phagolysosome, ~90% of the bacteria are successfully killed. However, the remaining ~10% can evade the intraphagolysosomal killing strategies and then use the cells as a niche to start replicating. Similar to non-professional phagocytes, the bacteria can eventually lyse the host cell and escape into the extracellular environment. The intracellular bacteria in all cell types are protected from immune cells as well as antibiotic treatment. Abbreviations: CRIg, complement receptor of immunoglobulin superfamily; SR, scavenger receptor; CR, complement receptor; FcR, Fc receptor.

Intracellular survival of *S. aureus* has been shown in vitro in almost every cell type, including epithelial cells, endothelial cells, osteoclasts, keratinocytes, and fibroblasts [23,24,25,26,27]. However, the infectious cycle caused by the bacteria depends on the cell type that is infected (Figure 1). In general, for the invasion of non-professional phagocytes, *S. aureus* employs a zipper-like mechanism that utilizes fibronectin-binding proteins A and B. These proteins attract fibronectin to the bacterial surface and this bacterial-protein complex is subsequently recognized by integrin α5β1, leading to the internalization of staphylococci in non-professional phagocytes [28]. Several additional staphylococcal surface proteins have been shown to promote internalization in mammalian cells, such as clumping factor A (ClfA), iron-regulated surface determinant B (IsdB), and efflux pump tet38 [28]. *S. aureus* can escape from the endosome into the cytosol of the host cell [29]. The bacteria can replicate inside the cytosol, eventually causing cell lysis and escaping into the extracellular environment (Figure 1, left panel). Various bacterial mediators have been suggested to play a role in this escape and survival mechanism. The bacterial two-component system (TCS) accessory gene regulator (Agr), a quorum-sensing regulatory system, has been found to be a key mediator in the process of the endosomal escape of bacteria [30,31,32]. In HeLa cells, Agr is required for autophagy-mediated cytotoxicity and is essential for bacterial escape into the cytoplasm, intracellular replication, and host cell killing [32]. Importantly, this intracellular *S. aureus* survival mechanism was not observed with an Agr mutant strain. The Agr system is a strict regulator for phenol-soluble modulin (PSM)-α, and -β production [33]. Using an elegant method of *S. aureus* detection in the cytosol, this study showed that PSM-α is crucial for the destruction of the endosomal membrane in epithelial and endothelial cells [34]. An intracellular role of PSMs is supported by the fact that serum lipoproteins can protect against PSM-induced host cell lysis, both in vitro and in vivo [35,36]. Thus, Agr and PSMs play important roles in the intracellular survival of *S. aureus* in non-professional phagocytes. However, evidence for intracellular survival in these cell types has only been shown in vitro and there has rarely been any direct visualization of staphylococci persisting in non-professional phagocytes in animal models of infection or in tissues dissected from infected patients.

## 3. The Infectious Cycle of *S. aureus* in Professional Phagocytes

Intracellular survival has also been observed in professional phagocytes such as neutrophils and macrophages [37,38]. There are important differences between these immune cells. Neutrophils are bone marrow-derived, short-lived, and mostly found in the bloodstream, although very limited numbers can be found extravasated in the peripheral tissues. Neutrophils are typically the first cells to arrive at the site of infection and have an incredible capacity for killing bacteria [39]. Macrophages, on the other hand, populate many different tissues and body cavities in large numbers. In homeostasis, macrophages can be derived from yolk-sac or fetal liver progenitors and are self-maintained by local proliferation or by bone marrow-derived monocytes. Upon inflammation, the turnover of macrophages is primarily monocyte-dependent, as monocytes are recruited and differentiate into macrophages. Depending on the tissue they populate, macrophages have very different gene expression profiles and can exert specific cellular functions such as clearance of dead cells, antigen presentation, and coordination of responses to infection [40]. 

In 1956, a seminal study by David Rogers showed that upon bloodstream infection in rabbits, a small number of bacteria was not removed from the blood by macrophages in the liver, but was internalized by neutrophils [41]. These intracellular bacteria were predicted to be responsible for the large wave of dissemination later in infection. Since then, others have shown that live *S. aureus* can survive in both mouse and human neutrophils and have proposed the “Trojan Horse” theory of dissemination by these immune cells [42]. Intravenous infection of *S. aureus* in mice leads to the uptake of bacteria by neutrophils that carry them in the blood for hours [43]. Isolated neutrophils from an infected animal were sufficient to cause full-blown disease in healthy animals [37]. Furthermore, patients with normal neutrophil counts may be more susceptible to bacterial dissemination compared to patients with reduced neutrophil counts [44]. Although neutrophils have been demonstrated to enable the persistence of *S. aureus* during infection, eventually leading to bacterial escape, there is limited experimental in vivo evidence that replication occurs inside these cells [37].

In contrast to neutrophils, there is an increasing body of in vivo evidence that intracellular replication occurs in macrophages. During bloodstream infection, the vast majority of staphylococci are taken up in the liver by the largest population of resident macrophages in the body, called Kupffer cells (KCs). KCs are a self-sustaining population of macrophages strategically situated within the liver sinusoids and in direct contact with the blood circulation [45]. In contrast to many other tissue macrophages and neutrophils, KCs are able to catch bacteria directly out of the bloodstream and overcome the high shear forces of blood flow [46]. KCs are crucial cells during bloodstream *S. aureus* infections, and KC depletion leads to massive, sustained bacteremia, and a greatly increased mortality due to *S. aureus* infections. Since KCs can kill ~90% of the injected inoculum, they are regarded as an important initial immune bottleneck during staphylococcal bloodstream infections, whereas neutrophils are essential at later time points [47]. Various studies have shown that not all staphylococci succumb to the intracellular bactericidal phagolysosome of KCs, and high-resolution intravital microscopy in combination with replication-reporter bacteria showed that staphylococci can replicate within these cells over time [15,19,20,21,22]. 

Using intravital microscopy in living animals, it was shown that *S. aureus* replicates in approximately 10% of infected KCs. Microcolonies of approximately 50–70 bacteria can be observed to lead to the destruction of the KC, which is followed by dissemination to various other internal organs, primarily the kidney (Figure 1, right panel). Mechanistically, *S. aureus* was found to replicate in phagolysosomes from KCs that did not produce sufficient reactive oxygen species (ROS) to kill the intracellular bacteria [15]. Interestingly, these replicating staphylococci induced α-toxin expression, which is released back into the circulation, thereby causing platelet aggregation, microthrombus formation, vascular occlusion, and organ dysfunction [48]. Even though α-toxin was upregulated by *S. aureus* in KCs, it remains to be determined which staphylococcal toxin is responsible for the lysis of Kupffer cells. Jorch et al. showed that after *S. aureus* escapes from the KCs, they relocate from the liver into the peritoneal cavity, where they get phagocytosed by Gata6^+^ peritoneal macrophages [49]. Similar to KC infections, *S. aureus* can persist inside the peritoneal macrophages and disseminate to organs in the peritoneal cavity, including the kidneys. Collectively, these data show that in the murine bloodstream infection model, *S. aureus* can infect and escape from multiple macrophage subtypes, ultimately seeding into the kidney, where it establishes classical abscesses 3–4 days post-infection. In a murine airway infection model, macrophages were also essential for the clearance of *S. aureus* since the loss of alveolar macrophages inhibited the killing of bacteria and significantly enhanced mortality [50]. Some studies have shown that *S. aureus* is actively taken up by alveolar macrophages, although it is currently unclear whether *S. aureus* replication occurs in this population of resident macrophages.

## 4. Intraphagolysosomal Evasion Strategies of *S. aureus*

**Figure 2:**
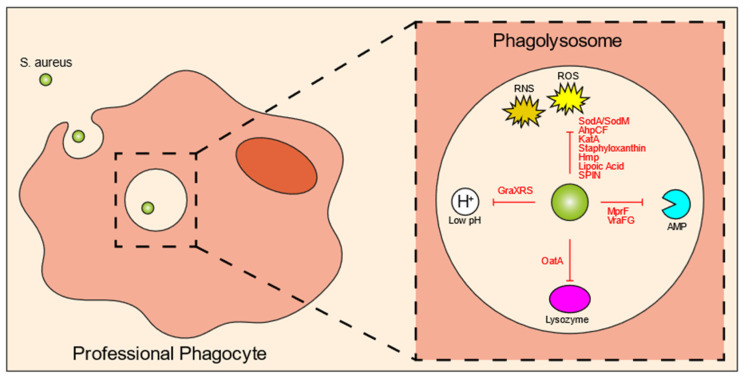
*S. aureus* intraphagolysosomal evasion strategies. Once inside the phagolysosome of professional phagocytes, the bacteria get exposed to various antimicrobial molecules designed to kill the bacteria. Co-evolution of host and pathogen has led to a variety of evasion molecules produced by *S. aureus* to counteract these phagolysosomal killing mechanisms. These mechanisms include evasion molecules against ROS and RNS, AMPs, lysozyme, and acidic pH. Abbreviations: ROS, reactive oxygen species; RNS, reactive nitrogen species; Sod, superoxide dismutase; AhpCF, alkyl hydroperoxidase reductase; KatA, catalase; Hmp, flavohemoglobin; SPIN, staphylococcal peroxidase inhibitor; AMP, antimicrobial peptide; MprF, multiple peptide resistance factor; OatA, O-acetyltransferase.

Unlike bacterial invasion observed upon the infection of non-professional phagocytes, professional phagocytes engage in a variety of phagocytic receptors to actively engulf and internalize bacteria via phagocytosis (Figure 1, right panel). During phagocytosis, the microbe is enclosed in a bacteria-containing phagosome within the phagocytic cell. These phagosomes undergo several maturation steps to ultimately become phagolysosomes, a process in which the interior of the vesicle progressively increases its acidity. Phagolysosomes contain a variety of antimicrobial peptides and degradative lysosomal enzymes, and are bombarded with ROS, all of which contribute to the killing of ingested bacteria [51]. The microbicidal activity of the phagolysosome is incredibly disruptive to most bacteria, and in order to establish an infection, many pathogens have evolved various strategies to neutralize or resist the effector components of the phagolysosome. Nonetheless, for *S. aureus*, there is some controversy on how these bacteria can overcome the phagosomal effector functions. Some literature suggests that the phagosomal maturation pathway is hijacked by *S. aureus* [22,52], while most papers found normal phagosomal maturation suggesting that *S. aureus* can withstand the harmful environment of the phagolysosome [3,15,21,34,53,54,55]. *S. aureus*, therefore, must overcome these defensive mechanisms in order to enable intracellular replication. Unsurprisingly, the bacteria express a plethora of evasion molecules inside the phagolysosome (Figure 2; Table 1). In this section, we will briefly highlight the most important mechanisms and some novel findings. For more information on this topic, there are some excellent reviews that have been previously published [17,28,56].

One of the most important killing mechanisms of the host cells is ROS, which is produced by NADPH oxidase (NOX2) via catalyzation of superoxide (O_2_−). Oxygen radicals have great antimicrobial activity, targeting various bacterial components, such as DNA, proteins, and the bacterial cell membrane. Nitrogen radicals can also be created by immune cells, termed reactive nitrogen species (RNS). It is not surprising that most *S. aureus* evasion molecules are directed against these molecules (Figure 2; Table 1). SodA and SodM are staphylococcal superoxide dismutases that directly eliminate ROS [57,58]. Similarly, alkyl hydroperoxidase reductase (AhpCF) and catalase (KatA) directly incapacitate peroxides and H_2_O_2_, respectively [59,60]. Staphyloxanthin is a carotenoid pigment with strong antioxidant properties [61]. For nitric oxide resistance, *S. aureus* can produce flavohemoglobin (Hmp), which acts like a denitrosylase [62]. ROS and RNS production can be inhibited by lipoic acid [63], and staphylococcal peroxidase inhibitor (SPIN) can bind to and block myeloperoxidase (MPO), which is the enzyme that converts H_2_O_2_ and chloride into hypochlorous acid [64]. 

A recent study by Leliefeld et al. using an in vitro fibrinogen gel model to increase the lifespan of neutrophils, showed that neutrophils limit staphylococcal growth in a pH-dependent manner [65]. Contrarily, in macrophages, it was shown that the intraphagolysosomal acidic pH is important for *S. aureus* to enable replication. This was orchestrated by GraXRS, which has been shown both in vitro as well as in vivo [66,67]. Similar to survival inside non-professional phagocytes, SigmaB could play a role in intraphagosomal survival, because a wild-type *S. aureus* strain showed increased resistance to acidic pH and hydrogen peroxide, when compared to a SigmaB-inactivated mutant in vitro [68]. In line with this observation, the SigmaB activating protein RsbU has been shown to be necessary for the intracellular growth of *S. aureus* within the phagolysosomes of THP-1 macrophages and HUVEC cells [69]. Various host cell-derived enzymes are released into the phagolysosome, among which are proteases. Lysozyme, which can cleave bacterial peptidoglycan and subsequently induce bacterial cell lysis, is particularly harmful. In response, *S. aureus* produces O-acetyltransferase (OatA) that acetylates peptidoglycan so it can no longer be targeted by lysozyme [70,71]. Finally, there are other antimicrobial peptides released into the phagolysosomal environment, many of which are positively charged. Multiple peptide resistance factors (MprF) of *S. aureus* catalyze a reaction that attaches a positively-charged lysine to the negatively-charged lipids of the bacterial membrane, thereby reducing interaction with antimicrobial peptides, defensins and protegrins in particular [72,73,74]. VraFG is a transporter of antimicrobial peptides and helps with resistance against cationic antimicrobial peptides [75]. 

Not all studies suggest that *S. aureus* replicates within the phagolysosome. Kubica et al. reported that *S. aureus* escaped into the cytosol of macrophages using α-toxin, where they replicated for a few days followed by host cell lysis. Several *S. aureus* factors were seen to be important for this survival, including SigmaB and Agr [76]. Another study suggested that the *S. aureus* USA300 strain can disturb normal phagolysosome formation and can sense the intraphagolysosomal pH in order to escape into the cytosol and commence intracellular replication [21]. Staphylococcus-produced PSMs have been shown to be important for killing neutrophils after phagocytosis. These toxins are actively upregulated by the bacteria within the phagolysosome by Agr and the stringent response system, and both systems appear to be crucial for the intracellular production of PSMs and escape from neutrophils [77,78].

## 5. Bacterial Specificity of the Intracellular Reservoir

Collectively, research has shown that *S. aureus* can persist and replicate in multiple cell types, maintaining the bacterial population during infection, and leading to the dissemination of bacteria throughout the body. Commensal bacteria are typically cleared by host phagocytes after infection, whereas non-aureus staphylococci can persist in macrophages but do not replicate [79]. Co-infection of *S. aureus* and commensal bacteria only led to the proliferation of *S. aureus* within KCs [80]. The commensal bacteria augment *S. aureus* infection by acting as a sink for ROS production, thereby increasing the chance that *S. aureus* can initiate replication inside KCs [81]. Many other bacterial, fungal, or parasitic species have been shown to use circulating phagocytes as an opportunity for dissemination, such as *Mycobacterium tuberculosis*, *Listeria monocytogenes*, *Leishmania*, and *Cryptococcus neoformans* [82,83,84,85].

The ability to persist and replicate intracellularly might be *S. aureus* strain specific. For example, where the Newman strain has been shown to be acid sensitive and is cleared in an intracellular infection [86], the more virulent USA300 strain can survive in phagocytes and has even been suggested to require acidification of the phagolysosome for persistence [67]. In a study where different *S. aureus* strains (Cowan I, 6850, Novel, Wood 46, BS 507, BS 513, BS 800, BS 890, BS 891, and RN4220) were tested in vitro with different cell lines (HEK-293, HeLa, and EA.hy926 cells), it was observed that all strains survived inside fully acidified vesicles except for *S. aureus* 6850, which was present in vesicles with reduced acidification [55]. Xiong et al. compared genotypes of persistent *S. aureus* strains with strains associated with rapid clearance in the blood and showed that persistence was associated with overexpression of Agr, staphylococcal cassette chromosome mec type II, clonal complex 30, and spa 16 [87]. Hence, only some *S. aureus* strains have the unique ability to persist and grow inside host phagocytes. As outlined above, various virulence mechanisms have implicated the ability of *S. aureus* to withstand the innate defenses of neutrophils and macrophages, even though not all studies are in agreement. The discrepancy between studies could be due to staphylococcal strain variation, use of distinct cells and or cell lines, and methodological differences. It should also be noted that in vitro generated data might be far from the actual in vivo survival mechanisms of *S. aureus* within macrophages. Nevertheless, it is safe to assume that there is a redundancy in evasion strategies that provides the bacteria time to adapt to the intraphagolysosomal environment to facilitate the intracellular reservoir.

## 6. Intracellular Persistence Expedites Antibiotic Tolerance

Antibiotic resistance is a continuous process, and indeed, there have been MRSA species identified in the population that are macrolide-resistant [88] or vancomycin-resistant [89]. The intracellular aspect of *S. aureus* infections certainly impedes antibiotic efficiency and may even assist in acquiring antibiotic tolerance. The antibiotic must reach the bacteria within the cell, and depending on where the bacteria reside, this requires passing the cell membrane once if the bacteria are in the cytosol, or twice if the bacteria remain in the phagolysosome. The fact that antibiotics cannot reach intracellular bacteria has already been described decades ago in vitro [90], and is shown to be an issue for antibiotic treatment of *S. aureus* infections. Indeed, treatment with antibiotics is far from ideal, with failure of treatment in up to 45% of patients [91]. Some classes of antibiotics are known to poorly penetrate cells, such as β-lactams [92] and aminoglycosides [93]. It is, therefore, not surprising that in vitro studies showed that β-lactams kill intracellular bacteria less efficiently than extracellular bacteria in human THP-1 macrophages [94]. 16 different types of antibiotics from 7 pharmacological classes were used in this study and showed that all molecules except for macrolides decreased the bacterial burden inside cells in a concentration-dependent manner. However, the intracellular activities of all antibiotics tested decreased compared to the extracellular activity, and only a few of the tested compounds had an intracellular bactericidal effect. As for vancomycin, its activity inside cells was greatly impaired when compared to extracellular killings, regardless of the concentration used [95]. Not only is passing through membranes important for killing intracellular bacteria, but the antibiotic must also be able to withstand acidic pH to kill bacteria inside the phagolysosome. It has been observed that an acidic environment decreases the intracellular concentration of gentamicin, macrolides, and quinolones [93]. Interestingly, the cellular concentration of β-lactams, and more prominently, rifampicin, were enhanced in an acidic medium, although this did not improve the intracellular activity of these antibiotics. Notably, acidic pH also affects antibiotic susceptibility to bacteria, as it has been shown that intracellular MRSA, which resists the activity of β-lactams extracellularly, regains sensitivity to β-lactams and vancomycin within the intracellular compartment [96]. Nevertheless, intracellular antibiotic activity remains to be a challenge. An in vitro study by Krut, Sommer, and Kronke showed that only rifampicin was able to eradicate intracellular *S. aureus* from murine keratinocyte (PAM212) and fibroblast (mKSA) cell lines [97]. Some tested antibiotics, including azithromycin, clindamycin, and linezolid, merely controlled extracellular bacteria, while *S. aureus* persisted intracellularly. Once the antibiotics were removed from the medium, *S. aureus* was able to regain its cytotoxicity and escape from these cells. Taken together, antibiotics need to be able to penetrate sufficiently into cells and withstand the bactericidal phagolysosome in order to kill intracellular bacteria. Even though the bacteria might regain susceptibility to certain antibiotics, the intracellular niche by and large creates opportunities for developing antibiotic resistance, especially when the antibiotic concentration within cells remains below minimum inhibitory concentration (MIC) levels [30].

*S. aureus* can change its phenotype to grow subpopulations that are considered to be more antibiotic-tolerant. These small colony variants (SCVs) are characterized as non-pigmented and non-hemolytic, are slow-growing, and have a colony size of approximately 1/10 of the normal *S. aureus* colonies [98]. SCVs can be auxotrophs, which is primarily due to mutations in electron transport as shown for menadione- and/or hemin-auxotrophs, or mutations in thymidylate biosynthesis, all of which may be a consequence of the downregulation of citric acid cycle activity [99]. SCV infections have been linked to relapsing or persistent infections in various diseases, such as cystic fibrosis and osteomyelitis [100,101]. SCVs have been demonstrated to be a highly antibiotic tolerant subpopulation of *S. aureus*, particularly because of their slow growth rate. As bacteriostatic antibiotics depend on obstructing bacterial cell growth for their function, this type of treatment is useless for SCV treatment, because the bacteria are already arrested at their cell growth. Bactericidal antibiotics on the other hand aim to cause cell death by interrupting active processes, such as peptidoglycan synthesis for β-lactams [102], translation for aminoglycosides [103], and DNA replication for fluoroquinolones [104]. However, because these processes are greatly reduced in SCVs, the bacteria can tolerate the antibiotics in their environment. Typically, SCVs only arise on a culture plate after 72 h, which is explained by mutations often observed in the Agr system to facilitate a non-hemolytic phenotype [105]. While other studies show that SCVs occur randomly without the involvement of Agr or Sae [106]. These mutations are reversible however, restoring SCVs to a fully cytotoxic wild-type phenotype, as has been shown in vivo where SCV-infected mice showed dissemination of bacteria in the spleen and kidney that were of wild-type phenotype [98].

Recently, Rowe et al. demonstrated that *S. aureus* remaining inside macrophages can acquire antibiotic tolerance to multiple antibiotics in the presence of rifampicin in a ROS-dependent manner [107]. The bacterial tricarboxylic acid (TCA) cycle was inhibited by ROS, leading to decreased respiration and ATP generation by the bacteria. Similar to SCVs of *S. aureus*, these persister cells have a significantly reduced metabolism, which is not compatible with antibiotics and allows the bacteria to actively exploit this antibiotic tolerance. The same group demonstrated that a variety of clinical *S. aureus* variants all acquired antibiotic tolerance to a similar extent when they were phagocytosed by activated macrophages, even though the ability of these strains to form a persister phenotype in vitro varied greatly [108]. Nevertheless, it was demonstrated that antibiotic tolerance was generated in a ROS-dependent manner in the spleen during a murine systemic infection model [107]. Host-derived peroxynitrite, the reaction product of hydrogen peroxide and nitrite, were found to be the main mediators for inducing this tolerance. In this study, the authors hypothesized that the bacteria reached a persister phenotype in a ROS-dependent manner. The influence of ROS on antibiotic tolerance is supported by studies showing that reduced phagocytic burst in macrophages increased antibiotic efficacy against *S. aureus* and that improved antibiotic function was observed when bacteria were engulfed in polymorphonuclear (PMN) cells of patients suffering from granulomatous disease [109]. In KCs, ROS production varies greatly between individual cells, and even between phagolysosomes within a single KC [15]. It is tempting to speculate that intracellular *S. aureus* may start replicating in phagolysosomes with low ROS activity and persister induction could occur in phagolysosomes with high ROS production. However, it remains to be investigated what percentage of staphylococci residing in the phagolysosomes of KCs develop into persisters and induce antibiotic tolerance. 

In summary, there are several factors contributing to the improved antibiotic resistance of intracellular *S. aureus*. First, antibiotics might not be able to reach the bacteria through the cell membrane. Second, antibiotic activity may be altered because of the intraphagolysosomal environment within macrophages. Third, over time, bacteria inside macrophages may change their phenotype to an SCV-like state, which is tolerant to antibiotic action. Finally, ROS inside phagolysosomes may induce a cell metabolism arrested state that, similar to the SCV phenotype, is antibiotic tolerant. Finding ways to reach the intracellular bacteria and kill them when they are in an inactive state is extremely important to counteract *S. aureus* infections.

## 7. Treatment Options to Manage Intracellular *S. aureus* Infections

**Figure 3:**
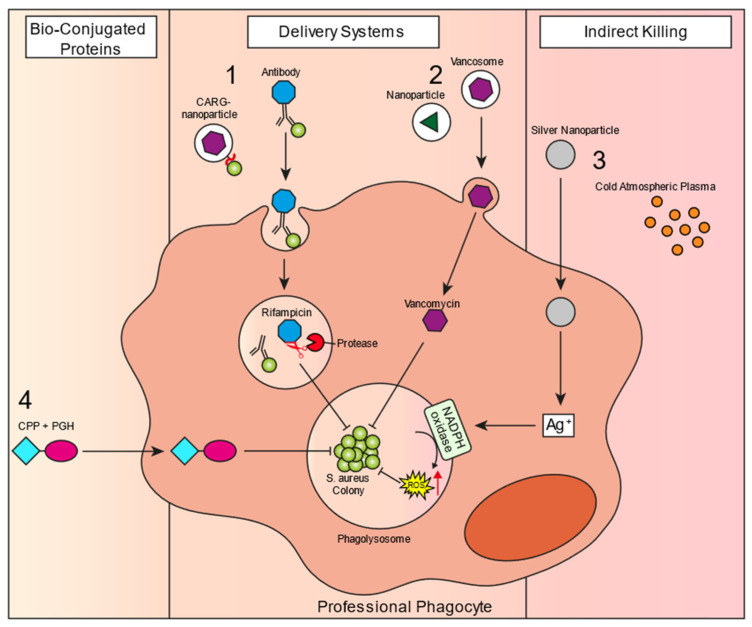
Novel treatment strategies to reach the intracellular reservoir of *S. aureus*. Schematic of different therapeutics that can penetrate host cells. **1.** *S. aureus*-targeted delivery: antibody conjugated to antibiotic—a specific *S. aureus* antibody binds extracellular bacteria and is taken up into the phagolysosome with the bacteria. Inside the phagolysosome, host-derived proteases can cleave off the rifalogue conjugated to the antibody, activating it, which can then attack the intracellular bacteria; CARG-nanoparticles—a specific peptide based on phage-displayed peptide libraries conjugated to vancomycin-containing nanoparticles. Similar to the antibody approach, it targets *S. aureus* and can reach the intracellular reservoir. **2.** Nanoparticle/liposome delivery system: antibiotics can be taken up inside nanoparticles or liposomes, which can fuse with the cellular membrane to deliver the encapsulated drug intracellularly. **3.** Indirect killing: silver nanoparticles deliver silver into the host cell cytoplasm, which in turn activates NADPH oxidase to induce ROS production, leading to improved killing of the intracellular reservoir. Cold atmospheric plasma can directly act upon NADPH oxidase to stimulate ROS production. **4.** Bio-conjugated proteins: cell penetrating peptides (CPPs) are molecules that can penetrate the host cell to deliver the attached peptidoglycan hydrolase (PGH).

In the last decade, a large number of new studies have identified treatment options that show promising activity against intracellular *S. aureus*. However, many of these studies have only been performed in vitro, so there is not much indication of their success in a clinical context. There are some recent reviews that give an overview of new compounds that have been developed over the last years [110,111,112]. In this section of the review, we summarize the most promising novel antibiotic strategies that specifically target the intracellular MRSA reservoir, which have shown in vivo effectivity or are currently being tested in clinical trials. We have divided the new treatment options into three different classes: delivery systems, conjugated bioproteins, and indirect killing mechanisms (Figure 3). 

### 7.1. Delivery Systems

To reach the intracellular reservoir of *S. aureus*, various delivery formulations have been developed that induce phagocytic uptake of the particle and, in turn, deliver the encapsulated antibiotic to kill the intracellular bacteria. These systems include various nanoparticles and liposomes that have been developed to deliver antibiotics non-specifically to the intracellular compartment of the host cells or have *S. aureus* specificity to reach the intracellular niche.

#### 7.1.1. Intracellular Delivery

Numerous studies have reported the development of nanoparticles that show intracellular effectivity against bacterial infections. Nanoparticles have great potential to encapsulate and carry drugs to intracellular bacteria and were already demonstrated to have treatment efficacy against well-known intracellular bacteria, such as *Listeria monocytogenes* and *Salmonella* spp. 30 years ago [113,114]. Like nanoparticles, antibiotic-loaded liposomes are another promising class of antimicrobial formulations that enhance intracellular antimicrobial efficacy against *S. aureus* infections. The efficacy of liposomes has been shown over a decade ago in a study that used gold nanoparticle-stabilized liposomes containing vancomycin as a compound against MRSA and showed improved inhibition of bacterial growth compared to free vancomycin [115]. As the bacteria reside in the acidic phagolysosomal environment, a promising approach was shown to be pH-responsive lipid-dendrimer hybrid nanoparticles (LDH-NPs), which were used to deliver vancomycin [116]. These LDH-NPs showed significantly improved killing of intracellular MRSA in HEK 293 cells in vitro. Complete eradication of MRSA was found when cells were treated with LDH-NPs, whereas millions of bacteria remained when treated with vancomycin alone. Importantly, the nanoparticles showed no toxicity towards HEK 293 cells, as none of these cells stained positive for live cells, indicating Hoechst 33342 staining. Additionally, another study had a similar approach with pH-responsive liposomes, which contained quaternary lipid (QL) and oleic acid (OA) in the membrane, which together formed a complex structure [117]. These QL-OA-complexes on the liposomal surface change the composition of acidic pH, creating pores and leading to drug release. Treatment with QL-OA-liposomes had a significantly improved antibacterial effect compared to vancomycin alone, both in TPH-1 macrophage and HEK293 cell lines in vitro. A similar effect was observed in a skin infection mouse model in vivo. Together, these results show that pH-responsive nanoparticles might be a promising new strategy to treat intracellular infections. To deplete KCs, clodronate-loaded liposomes were the method of choice for many years before ingenious genetic tools became available. Furthermore, it has been shown that upon intravenous injection, 70% to 80% of liposomes concentrate in KCs [118]. This propensity for KCs to take up liposomes inspired us to use liposomes containing vancomycin or vancosomes to precision-target the intracellular reservoir of *S. aureus* within KCs [15]. The efficacy of vancosomes was 30-fold higher than treatment with vancomycin alone, which was not able to reach the intracellular staphylococci. Intravital microscopy showed that vancosomes co-localized with MRSA-containing KCs and increased intracellular killing was observed. This was sufficient to prevent liver damage and bacterial dissemination to the kidneys. Importantly, upon the termination of the antibiotic treatment, the staphylococci escaped from the intracellular niche and caused dissemination to the kidney in the vancomycin control group, which was not seen when the intracellular reservoir was eradicated by vancosomes. As vancosomes have shown their efficacy in vivo, this might be an exciting strategy to target the intracellular bacterial reservoir in patients. Finally, a study by Li et al. generated membrane-encapsulated, antimicrobial-conjugated nanoparticles (Me-ANPs) consisting of self-assembling triclosan, and ciprofloxacin conjugates within membranes isolated from J774.A1 macrophages [119]. These Me-ANPs were found to specifically be taken up by *S. aureus*-infected macrophages, as the negative charge of the nanoparticles could bind to positive patches of infected macrophages that were lysozyme-rich. Furthermore, Me-ANPs showed significantly improved killing capacity of intracellular *S. aureus* in J774.A1 macrophages in vitro compared to standard NPs or free ciprofloxacin. Me-ANP treatment was also significantly more effective in vivo, both in an acute peritoneal infection model as well as in a mouse organ infection model. This shows that coating nanoparticles with macrophage membranes could enhance the delivery potential of antibiotics.

#### 7.1.2. *S. aureus*-Targeted Delivery

Investigators from Genentech took a completely novel approach to target the intracellular reservoirs. They developed an antibody-antibiotic conjugate (AAC) where an anti-staphylococcal wall techochoic acid (WTA) monoclonal antibody was conjugated to a rifalogue [14]. The antibiotic rifalogue can be liberated from the antibody when it is cleaved off by intracellular cathepsins and does not have an antibiotic function when still attached to the antibody. The antibody is *S. aureus* specific, and binds extracellular bacteria, leading to internalization while still attached to the bacteria. When the antibody-antibiotic-bacteria complex is phagocytosed, intraphagolysosomal cathepsin liberates the antibiotic that can subsequently kill the bacteria. This is a very promising method to kill hard-to-treat intracellular infections as it showed great efficacy in staphylococcal bacteremia models [14], and this AAC is currently in a phase-one clinical trial [120]. In line with this, another more recent study used phage-displayed peptide libraries to select peptides with a high binding capacity to *S. aureus* [121]. In vivo screening confirmed the binding of the peptides to bacteria in *S. aureus* infected lungs. Next, an identified peptide of interest, CARGGLKSC (CARG), was conjugated to vancomycin-loaded nanoparticles. In a murine lung infection model, the nanoparticles were shown to specifically co-localize in *S. aureus* infected lungs, whereas no nanoparticle localization of Pseudomonas infected lungs was observed. Furthermore, 100% survival of CARG-nanoparticle treated mice was observed compared to 33% survival after an equivalent dose of vancomycin. One great advantage of these targeted delivery systems is that only the specific microbe is targeted, while broad-spectrum antibiotics indiscriminately kill both pathogens and commensals. The latter could result in dysbiosis of the gut microbiota, which has been associated with *Clostridium difficile* infections and diseases such as inflammatory bowel disease, obesity, and atherosclerosis. However, the intracellular specificity of these approaches could also be considered a disadvantage as it does not kill MRSA extracellularly, nor does it eliminate biofilm-associated infections. Additionally, in patients with low phagocyte levels, such as cancer patients that receive chemotherapy, which is a known risk factor for staphylococcal infections, this approach may be ineffective.

### 7.2. Bio-Conjugated Proteins

Similar to nanoparticles and liposomes, bio-conjugation provides a delivery strategy for existing antibiotics into the host cells to kill the intracellular staphylococcal reservoir. A few bio-conjugated proteins have been described in the literature, of which one has shown in vivo effectivity. In this study, the authors looked into peptidoglycan hydrolases (PGHs), such as lysostaphin, for bio-conjugation [122]. Peptidoglycan hydrolases are enzymes that can cleave the peptidoglycan of the bacterial cell wall, inducing lysis. After screening for various cell-penetrating peptides (CPPs) and PGHs, bio-conjugation of lysostaphin (LST) to either trans-activator of transcription (TAT) or penetratin showed the most promise, as improved intracellular killing of different *S. aureus* strains (Newman, Cowan, and USA300 JE2) was observed in vitro in three eukaryotic cell lines (A549, MG-63, and 3T3-L1). No detectable CFU counts were observed for these bio-conjugated proteins after 4 h of treatment compared to considerable viable CFUs (~10^4^ CFU/mL) after treatment with LST alone. The authors also showed that a variety of different CPG-PGH combinations worked in synergy to kill bacteria, with decreased intracellular bacterial counts, weight loss, and abscess volume in a murine abscess model compared to the individual CPG-PGH molecules. These CPG-PGH cocktail therapies are a promising treatment option for intracellular infections.

### 7.3. Indirect Killing Mechanisms

Rather than delivering an antibiotic into the cells, indirect killing mechanisms are strategies to boost host-cell killing capacities against *S. aureus*. Two approaches discussed below activate ROS production by macrophages. Other approaches led to the activation of intracellular receptors of innate immunity NOD2, which detected fragments of peptidoglycans-muramyl peptides, including those from *Staphylococcus aureus*. Inducing ROS production could be a very valuable antibacterial strategy, as the literature has shown that phagolysosomes with a reduced ROS capacity are most vulnerable to harboring the intracellular staphylococcal reservoir. Therefore, these approaches could prove to be useful tools for antibiotic treatment, either on their own or in combination with bacterial-targeted approaches.

#### 7.3.1. Silver Nanoparticles

Metal nanoparticles have multiple antimicrobial properties that could be useful for intracellular infections, especially because these combined properties decrease antibiotic resistance opportunities for the bacteria. Silver nanoparticles (AgNPs) release Ag+ ions that can both disrupt electron transport and induce ROS production, which in turn harms the bacteria [123]. These AgNPs have been shown to kill 100% of extracellular bacteria as well as 76% of intracellular *S. aureus* within osteoblasts. The intracellular killing was at similar rates to the rifampicin treatment, whereas no intracellular killing was shown with gentamicin treatment alone. A clinical trial (NCT04431440) took sputum and blood samples of 75 critically ill patients and found 55.3% occurrence of MRSA and 12.7% occurrence of VRSA in the samples [124]. *S. aureus* bacteria were isolated from the samples and tested on culture plates with AgNP-discs, which showed effective inhibition of growth for both MRSA and VRSA variants. If efficacy is shown in vivo, AgNPs may provide a useful tool to combat *S. aureus* infections.

#### 7.3.2. Cold Atmospheric Plasma

Cold atmospheric plasma (CAP), electrical discharged gas, has antimicrobial properties, which a study by Duchesne et al. investigated [125]. It was found that CAP treatment increased intracellular ROS in RAW 264.7 macrophages irrespective of whether the cells were infected with *S. aureus*. CAP significantly reduced intracellular MRSA USA300 infection in macrophages over 7 h of infection compared to an untreated control. However, bacterial killing by CAP was only improved by approximately 45% compared to the untreated bacteria, and treatment was initiated prior to bacterial infection, which is not relevant in a clinical situation. The authors concluded that CAP increased ROS production by macrophages, leading to an increased killing capacity. In a recent clinical pilot study, CAP was tested on patients with mild to moderate atopic dermatitis [126]. CAP-treated patients showed significant improvement in the clinical score for the severity of the disease. Additionally, a microbiome analysis was performed with 16s-RNA sequencing before and after treatment, which showed a significant decrease in *S. aureus* prevalence in the skin microbiome, compared to sham patients. Together, these studies show that CAP is safe to use on patients, but the intracellular effect of this compound must be examined further. This compound might prove to be useful in combination with other therapeutic strategies.

#### 7.3.3. Muramyl Peptides

Activation of intracellular NOD receptors to potentiate the killing of intracellular *S. aureus* is another important avenue of research. Muramyl peptides present in the staphylococcal cell walls have been shown to increase the phagocytic and microbicidal activity of neutrophils, including ROS production [127]. The synergism of NOD2 receptor activation together with anti-staphylococcal immunoglobulin in patients with chronic sluggish furunculosis with identified antibiotic-resistant strains of staphylococcus made it possible to achieve remission in 99% of patients while using only anti-staphylococcal immunoglobulin. Remission was achieved in 40% of patients during six months of observation [127]. An explanation for the therapeutic effect of muramyl peptides in the treatment of diseases caused by antibiotic-resistant strains of staphylococcus may be an increase in the production of cyclooxygenase-2 (COX-2) [128] and the recruitment of neutrophils when exposed to muramyl peptides together with staphylococcus aureus lipoteichoic acid at the site of inflammation [129]. 

In summary, innovative targeting approaches are continuously in development, many of which show promising results for treating intracellular *S. aureus* infections.

## 8. Conclusions

It has become clear over the past decades that *S. aureus* has an intracellular aspect to its infectious cycle, leading to infections that are difficult to treat. Persistence in both non-professional as well as professional phagocytes has been suggested in an abundance of studies. Moreover, it has been shown in vivo that *S. aureus* can use liver KCs as a niche to replicate upon bloodstream infection, while hiding from other host immune cells as well as antibiotic therapy. This intracellular aspect of the bacterial infectious cycle could in turn stimulate adaptations in *S. aureus* that favor antibiotic tolerance. Thus, it is crucial to find alternate strategies to kill the intracellular staphylococcal reservoir. So far, several new therapeutic strategies have been suggested with variable, yet promising results. Initially, during bloodstream infection, 10% survival of the bacteria inside KCs is enough to enable the preservation of an intracellular reservoir; any potential antibiotic strategy must further reduce that survival rate. Likely, some combination of currently available antibiotics plus a novel therapeutic strategy will have optimal efficacy, as not only the intracellular, but also the extracellular bacteria need to be targeted. Finding the ideal possible combination is of high importance, as AMR remains a global health problem. The herein described strategies illustrate major steps in the right direction towards finding a suitable approach to deal with the intracellular staphylococcal reservoir.

## Figures and Tables

**Table 1:** *S. aureus* intraphagolysosomal evasion strategies. Bacterial evasion molecules against reactive oxygen species, acidic pH, enzymes, and antimicrobial peptides present in the host phagolysosomal environment. Abbreviations: ROS, reactive oxygen species; Sod, superoxide dismutase; AhpCF, alkyl hydroperoxidase reductase; KatA, catalase; Hmp, flavohemoglobin; RNS, reactive nitrogen species; SPIN, staphylococcal peroxidase inhibitor; MPO, myeloperoxidase; OatA, O-acetyltransferase; MprF, multiple peptide resistance factor; AMPs, antimicrobial peptides.

Intracellular Killing Mechanisms	*S. aureus* Evasion Molecule	Mechanism/Explanation	References
ROS	SodA and SodM	Incapacitate superoxide radicals	Karavolos et al., 2003; Das, Saha and Bishayi 2008 [57,58]
	AhpCF	Resists peroxides	Cosgrove et al., 2007; Mashruwala and Boyd 2017 [59,60]
	KatA	Resists H2O2	Cosgrove et al., 2007; Mashruwala and Boyd 2017 [59,60]
	Staphyloxanthin	Antioxidant	Pandey, Sahukhal and Elasri 2019 [61]
	Hmp	Resistance to nitric oxide	Nobre, Gonçalves and Saraiva 2008 [62]
	lipoic acid	Restricts ROS and RNS production	Grayczyk et al., 2019 [63]
	SPIN	Inhibits MPO	de Jong et al., 2017 [64]
Acidification	GraXRS	Senses low pH and allows resistance to acidic environment	Flannagan et al., 2018 [67]
Enzymes	OatA	Modifies lysozymal target	Bera et al., 2006; Shimada et al., 2010 [70,71]
Antimicrobial peptides	MprF	Resistance to defensins and protegrins	Kristian et al., 2003; Peschel et al., 2001; Peschel et al., 1999 [72,73,74]
	VraFG	Promotes resistance to cationic AMPs	Li et al., 2007 [75]
